# A Simplified Heat-Tolerance Evaluation System at the Pollen Development Stage in Rice (*Oryza* *sativa* L.)

**DOI:** 10.3390/plants15081253

**Published:** 2026-04-18

**Authors:** Saihua Chen, Yuhui Liu, Ning Xiao, Yan Sun, Luyao Zhang, Xiaofan Yi, Ming Xue, Aihong Li, Mingliang Xu

**Affiliations:** 1 Jiangsu Key Laboratory of Crop Genomics and Molecular Breeding, Agricultural College of Yangzhou University, Yangzhou 225009, China; chensaihua@yzu.edu.cn (S.C.);; 2 Institute of Agricultural Sciences for Lixiahe Region in Jiangsu, Yangzhou 225007, China

**Keywords:** rice, heat stress, heat tolerance, evaluation system, pollen development

## Abstract

Heat stress, particularly during the reproductive stage, poses a major challenge to rice production, as pollen development is highly sensitive to elevated temperatures. Accurate assessment of heat tolerance during this period is crucial for improving rice heat-stress tolerance but is hindered by asynchronous panicle development and imprecise staging. In this study, we identified a pair of near-isogenic lines, ZP15 and ZP17, which exhibited contrasting seed-setting rates under heat stress. We demonstrated that this divergence arises from differential tolerance during the pollen developmental stage, corresponding to a critical window (9–16 days before heading). Taking these lines as references, we established a reliable system that synchronizes developmental staging and quantitatively assesses heat-induced fertility loss. Validated using heat-tolerant N22 and heat-sensitive Wushansimiao, this system was applied to assess four conventional varieties and eight hybrids. Huanghuazhan and self-bred hybrids (Yangxianyou 912, Yangxianyou 903, and Yangxian 9A/P119-8) displayed high tolerance comparable to control varieties, whereas Yangdao 6 and multiple hybrids showed pronounced sensitivity. Collectively, this work provides a precise and reproducible framework for evaluating heat tolerance during pollen development, offering a valuable tool for accelerating the breeding of heat-resilient rice varieties.

## 1. Introduction

Extreme heat events driven by global warming are reducing crop productivity and threatening food security. According to the World Food and Agriculture Organization, heat stress has caused a 9.1% decline in global harvests [1]. Research from the International Rice Research Institute indicates that rice production decreases by ~10% with each 1 °C increase in minimum temperature during the growing season [2]. In China, the high-temperature indices during the rice production period in the middle and lower region of the Yangtze River have increased significantly [3]. It was predicted that the frequency, duration, and intensity of high-temperature events would continue to rise in Hubei, Anhui, Hunan, and central and northern Jiangxi. Furthermore, simulations using the CERES-Rice model predict that heat stress could reduce rice yields by up to 60% under future climate scenarios [4]. Together, these findings highlight that high temperature has become one of the most critical threats to rice yield, and ensuring stable rice production under extreme weather conditions presents a pressing global challenge.

Although rice growth can be adversely affected by extreme heat at all developmental stages, the reproductive development stage is particularly vulnerable and more likely to coincide with high-temperature events in agricultural production, leading to spikelet sterility and yield reduction. Wheeler et al. reported that short episodes of high temperature around anthesis had a greater impact on crop yield than a seasonal mean temperature increase of ~2 °C [5]. Severe spikelet sterility occurs when temperatures exceed 35 °C at anthesis for more than 1 h [6]. During the heading or reproductive stage, the grain yield is significantly reduced when the average daytime temperatures exceed 33 °C or nighttime temperatures exceed 32 °C [7,8]. High temperatures impair multiple processes in reproductive development, including gamete formation, flowering, fertilization, and grain filling, ultimately reducing yield [9,10,11,12,13]. During the gamete development period, pollen formation is particularly sensitive to high temperatures, which in turn markedly reduces the seed-setting rate in rice. Pollen formation is a continuous process consisting of four stages: somatic cell differentiation, meiosis, microspore formation, and pollen maturation/release. At the microspore stage, exposure to heat stress (39 °C during daytime/30 °C at night) results in complete loss of spikelet fertility, indicating that this stage is the most heat-sensitive phase of pollen development [14].

For screening heat-tolerant rice germplasms and conducting related genetic studies, an accurate evaluation system for heat tolerance is essential. Assessments targeting the reproductive stages remain challenging due to limited throughput and the complexity of developmental processes. Most studies focused on the flowering period, in which plants are subjected to heat stress at mid-heading or anthesis, followed by examination of pollen germination on the stigma or measurements of spikelet fertility (seed-set) [12,15,16]. In these cases, pollen development is nearly complete, and whether mature pollen grains can withstand high temperatures has been tested. By contrast, the earlier microspore formation stage prior to flowering was also examined using −1 to +1 cm Auricle Distance (AD) as a benchmark [14]. When approximately 10% of plants reached the heading date, rice was considered to have entered the meiosis stage and then subjected to heat treatment [17]. However, the timing of heat treatment is often broad and imprecise due to the inconsistent development of different panicles and florets. A precise and feasible heat-tolerance evaluation system covering the entire pollen developmental stage is still lacking.

The present study aims to address the lack of efficient evaluation tools for stage-specific heat-stress tolerance during pollen development in rice. To this end, we first intend to identify ideal materials for dissecting the critical pollen developmental stage associated with heat tolerance. Using these identified lines as references, we aim to establish a simplified and reliable evaluation system for heat-stress tolerance specifically targeting the pollen development period. We plan to validate this system using well-characterized reference materials and subsequently apply it to assess heat tolerance in diverse varieties and hybrids, ultimately providing a practical platform that lays a foundation for improving stage-specific heat tolerance in rice breeding programs.

## 2. Results

### 2.1. ZP15 Shows Higher Seed-Setting Rates than ZP17 Under High-Temperature Conditions

**Figure 1:**
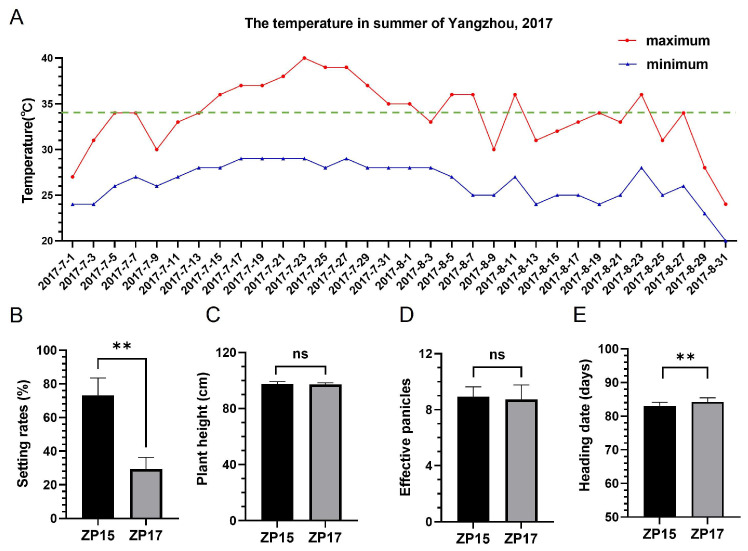
Comparison of agronomic traits in ZP15 and ZP17 in natural high-temperature field conditions. (**A**) Summer temperatures in Yangzhou, 2017. The red line represents the daily maximum temperature, and the blue line represents the daily minimum temperature. The green dotted line in the figure indicates 34 °C, which is the critical high-temperature threshold for rice during the reproductive stage. The horizontal axis denotes the dates. (**B**) Seed-setting rates, (**C**) plant height, (**D**) effective panicles, and (**E**) heading date data. The symbol “ns” indicates no significant difference (*p* > 0.05) and ** indicates an extremely significant difference (*p* < 0.0001) between ZP15 and ZP17 by Student’s *t*-test.

In 2017, a natural high-temperature climate occurred, compromising rice yields. Meteorological data from one website (https://www.tianqi24.com/) indicated that the high-temperature climate persisted into July and August in Yangzhou city. The average daily maximum temperature reached 37.56 ± 1.62 °C in mid-to-late July, exceeding the 34 °C threshold for rice during the flowering period (Figure 1A). Under these conditions, ZP15 and ZP17 exhibited a significant difference in seed-setting rates, with ZP15 at 73% and ZP17 at 28% (Figure 1B), which had not been observed previously. Importantly, there were no significant differences in plant architecture, including plant height and number of effective panicles (Figure 1C,D). The heading dates (HDs) of ZP15 and ZP17 were 82.73 ± 1.28 and 84.20 ± 1.26 days, respectively (Figure 1E). Although they showed an approximate 1.5 days difference in HD, most panicles of both lines were exposed to hot weather concurrently, and the divergence in seed-setting rates was likely due to heat stress.

**Figure 2:**
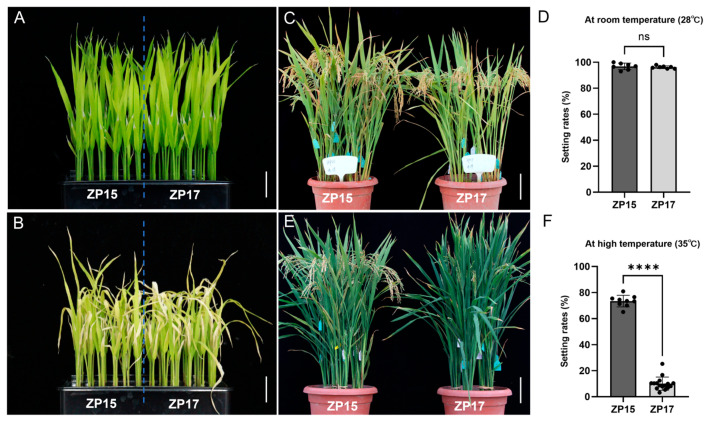
Heat resistance comparisons at the seedling and reproductive stages. Two-week-old seedlings and plants at the reproductive stage were subjected to heat stress treatment. (**A**) Seedlings before treatment; (**B**) seedlings after 48 h of treatment at 42 °C; (**C**) plant morphology and (**D**) seed-setting rates under artificial normal temperature conditions; (**E**) plant morphology and (**F**) seed-setting rates under artificial heat stress conditions. (**A**,**B**) bar = 2 cm; (**C**,**E**) bar = 15 cm. The symbol “ns” in (**D**,**F**) indicates no significant difference (*p* > 0.05) and the stars indicate an extremely significant difference (*p* < 0.0001) between ZP15 and ZP17 by Student’s *t*-test. The black dots represent the experimental data points collected in this study.

To test this hypothesis, ZP15 and ZP17 were subjected to controlled high-temperature treatments in a climate chamber. At the seedling stage, no significant difference was observed after 42 °C treatment for 48 h (Figure 2A,B). At the reproductive stage, plants were treated for two weeks before flowering. Under normal temperature conditions, there was no difference in seed-setting rates between ZP15 and ZP17 (Figure 2C,D). By contrast, significant seed-setting differences were observed in the panicles that headed within one week following the heat treatment. Those in ZP17 exhibited nearly a 90% reduction in seed-setting rate, whereas a rate close to 80% was maintained in ZP15 (Figure 2E,F). Collectively, these results demonstrate that ZP15 has stronger high-temperature tolerance than ZP17 specific to the reproductive stage.

### 2.2. High Temperature Affects Pollen Development in ZP15 and ZP17 Differently

**Figure 3:**
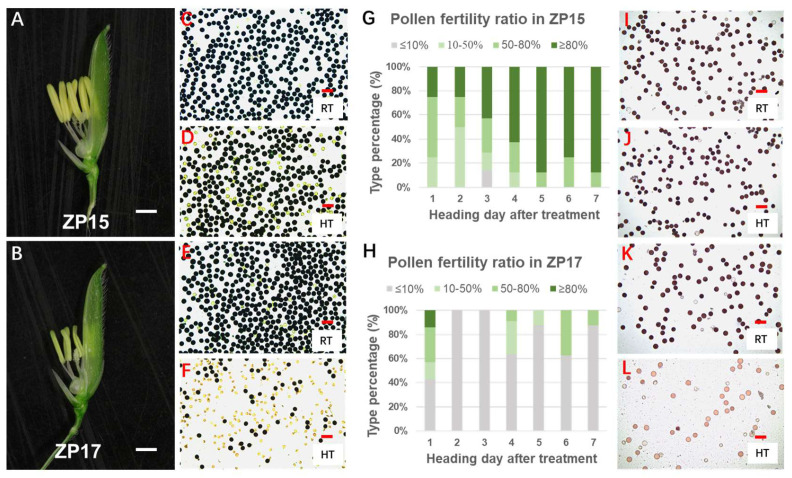
Pollen viability comparison under normal and high-temperature conditions. (**A**,**B**) Anther phenotypes of (**A**) ZP15 and (**B**) ZP17 after high-temperature treatment; bar = 1.5 mm. (**C**,**D**) The I_2_–KI staining results of ZP15 pollen at (**C**) room temperature and (**D**) high temperature. (**E**,**F**) The I_2_–KI staining results of ZP17 pollen at (**E**) room temperature and (**F**) high temperature. Fertile pollen grains, rich in starch, were stained deep black, whereas abortive pollen grains, lacking starch accumulation, remained unstained and appeared light yellow. (**G**,**H**) The I_2_–KI staining proportion of each floret was divided into four types: ≤10%, 10–50%, 50–80%, and ≥80%. The stacked bar charts show the percentage of each type in (**G**) ZP15 and (**F**) ZP17. The horizontal axis is the day number after 2-week heat treatment. (**I**,**J**) The TTC staining results of ZP15 pollen at (**I**) room temperature and (**J**) high temperature. Dark-red staining indicates good pollen viability, as viable pollen reduces colorless TTC to insoluble red formazan via active respiratory dehydrogenases; light-red or unstained pollen indicates poor or inactive viability, reflecting diminished or lost respiratory enzyme activity. (**C**–**F**,**I**–**L**) bar = 100 µm.

It has been reported that pollen development is highly sensitive to high temperatures. In 2017, a natural high-temperature climate coincided with the reproductive stage of ZP15 and ZP17, suggesting that their contrasting setting rate might have resulted from impaired pollen development under heat stress. To test this, we examined anther morphology and pollen development under both normal and high-temperature conditions. Under normal conditions, both florets of ZP15 and ZP17 displayed full, bright-yellow anthers. After high-temperature treatment, however, most ZP15 florets still produced normal anthers (Figure 3A), whereas ZP17 florets frequently developed atrophied, pale-yellow anthers (Figure 3B).

I_2_–KI staining further revealed that the proportion of stained pollen in ZP15 declined slightly after heat treatment (Figure 3C,D), while ZP17 showed a dramatic reduction (Figure 3E,F). To quantify pollen fertility, all florets in the panicles (heading within one week after heat treatment) were collected and subjected to I_2_–KI staining, and finally classified into four groups based on staining percentages: ≤10%, 10–50%, 50–80%, and ≥80%. In panicles heading at 1–4 days after treatment in ZP15, a large proportion of florets maintained high pollen viability (staining percentage > 50%), while for those heading at 5–7 days after treatment, the staining percentage of all florets was >50% (Figure 3G). In contrast, florets with low pollen viability (staining percentage < 10%) accounted for a large proportion in ZP17: up to 100% of florets in the panicles heading 2–3 days after treatment (Figure 3H). These pollen fertility patterns closely matched the final setting rate, indicating that the higher setting rate in ZP15 under heat stress is attributable to its stronger tolerance during pollen development.

TTC staining provided further support. ZP15 pollen retained stable staining under both normal and high-temperature conditions (Figure 3I,J), whereas the staining intensity was significantly reduced after heat treatment in ZP17 pollen (Figure 3K,L). Taken together, these results indicate that the superior heat tolerance of ZP15 primarily reflects its stronger viability during pollen development.

**Figure 4:**
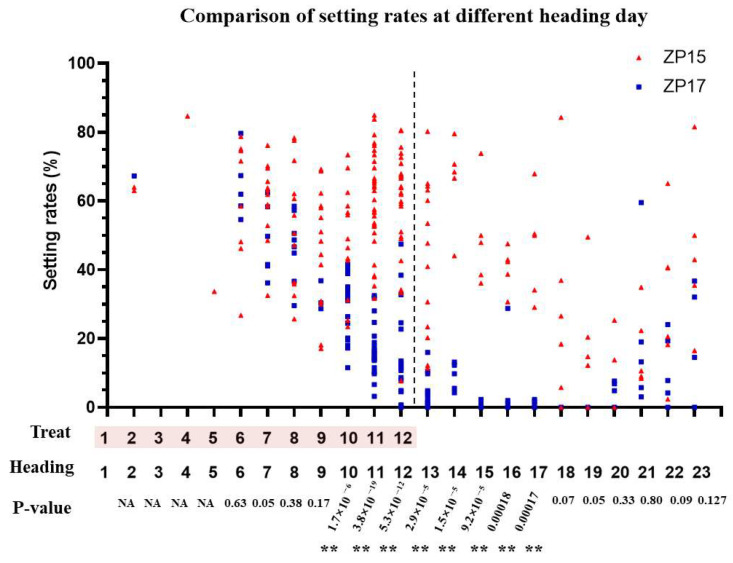
Comparison of setting rates in panicles heading on different days. The red triangles represent data from ZP15, and the blue squares represent data from ZP17; each symbol denotes seed-setting rate data from a single panicle. The horizontal axis indicates both the 12-day treatment period (numbers in pink) and the corresponding heading days during the treatment. ** indicates a significant difference between ZP15 and ZP17 (*p* < 0.01).

To pinpoint the critical window for their pollen developmental divergence, panicles at different pollen development stages were simultaneously exposed to high temperatures in a controlled climate chamber. The HD of each panicle was marked daily, and their final seed-setting rates were recorded. After heat treatment commenced, the panicles started to head sequentially. The final seed-setting rates of the panicles who exhibited heading from the 6th to 9th day exhibited a significant decrease day by day, yet no significant difference was observed between ZP15 and ZP17 (Figure 4, Supplemental Figure S1). Meanwhile, significant differences between ZP15 and ZP17 emerged in the panicles that headed from the 10th to the 17th day (Figure 4, Supplemental Figure S1). Thereafter, the setting rates of panicles from both ZP15 and ZP17 were significantly compromised. From these effective 10th-to-17th-day heading panicles, the critical time for their heat-tolerance variations can be extrapolated from the 9–16 days before heading, corresponding to the meiosis and microspore developmental stages of pollen [18,19].

### 2.3. Establishment of an Evaluation System for Heat Tolerance During the Pollen Developmental Stage

Leveraging the contrasting heat tolerance of ZP15 and ZP17 during the pollen developmental stage, we designed an accurate evaluation system for gamete-specific or pollen-specific heat tolerance using ZP15 and ZP17 as references.

Through preliminary temperature tests conducted on ZP15 and ZP17 (Supplemental Figure S2), we found that constant daytime temperatures of 38 °C and 41 °C inhibited heading, with most panicles remaining enclosed by flag leaves, resulting in delayed heading or even complete heading failure. In contrast, a constant daytime temperature of 35 °C provided sufficient stringency to distinguish the heat tolerance difference between ZP15 and ZP17. Therefore, a diurnal temperature regime of 35 °C (14 h light)/29 °C (10 h dark) was selected as the standard heat treatment condition. The temperature within the growth chamber was continuously monitored using a BIOVOICE Temperature Dataloggers T301 (Supplemental Figure S3).

**Figure 5:**
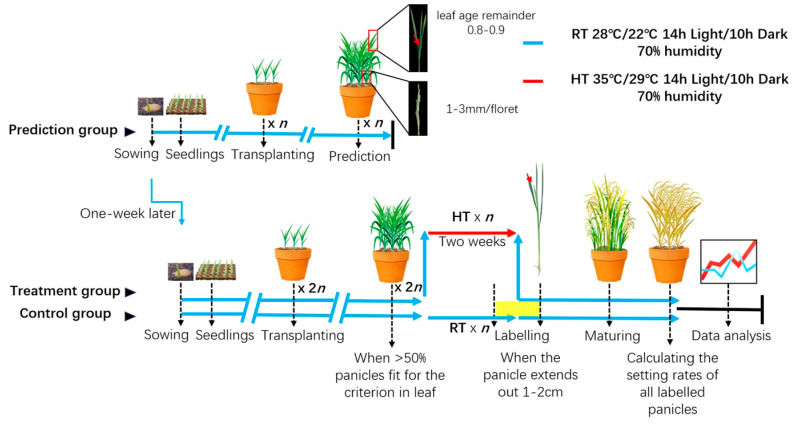
Schematic illustration of the evaluation system. Three groups are set in this system. The upper panel shows the processes of the prediction group. After sowing, one-month seedlings were transplanted into pots. If the last leaf was 1–2 cm out of the leaf sheath (leaf age remainder 0.8–0.9), their stems were peeled to observe the status of the meristems. If the panicle was developed and each floret in the panicle was around 1–3 mm in length, that meant the prediction group was approaching the right time for treatments [20], which suggests that the other two groups (the control and treatment group indicated in the lower panel) would be ready one-week later. The number (*n*) of pots for each tested line is around 6, with 3–4 plantlets per pot. The lower panel shows the processes of the control and treatment groups. The number (2n) of pots for each tested line is no less than 12, with 3–4 plantlets per pot. These lines were sown one week later than the prediction group. About one week after the prediction group reached the relevant developmental stage, if >50% panicles began to pull out of the last leaf, half of the pots were kept at room temperature (RT) as the control group and the other half were transferred to high-temperature (HT) conditions for a 12-day treatment as the treatment group. After treatments, the treatment group was moved out of the heat condition and continue to grow in the room-temperature (RT) condition. The labeling of heading panicles started on the 10th day of the treatment and lasted until the 3rd day after the treatment (in yellow). After maturing, the setting rates of all the labeled panicles were calculated and the data was compared between the two groups. If no compromise or no significant divergence among different varieties was observed under normal conditions, the heat tolerance among different materials could be assessed directly by seed-setting rates under HT condition. n indicates the number of plots; at least 6 pots were needed. The red arrow represents the high-temperature treatment condition, while the cyan arrows indicate the normal temperature conditions.

To precisely determine the initiation of pollen development, we referred to the criteria established by Prof. Wang [20], using a leaf age remainder of 0.8–0.9 and a spikelet length of 1.0–1.9 mm as morphological markers. Given that pollen development spans approximately 12 days, the heat stress duration was set to 12 days to cover the entire pollen developmental process. Furthermore, based on observations of ZP15 and ZP17 panicles at different heading stages, we identified the critical heat-sensitive window as 9–16 days before heading. Accordingly, panicles were labeled and sampled within 3 days before and after the end of the heat treatment period (indicated by the yellow rectangle in Figure 5).

To accurately predict the developmental stage, a separate prediction group was established. To minimize the influence of growth chambers on rice growth and development, a room-temperature control group was established simultaneously with the high-temperature treatment group. Thus, the system included a total of three groups.

The prediction group was sown one week earlier than the other two groups and served to accurately determine the onset of pollen development. When the potential final flag leaf extended 1–2 cm from the sheath, corresponding to a leaf age remainder of 0.8–0.9 [20], main stems were dissected for further confirmation (Figure 5, upper panel).

The control and treatment groups were sown and transplanted simultaneously, one week later than the prediction group. When the prediction group reached the target stage, the other two groups were expected to fully expand their final leaf within one week. Once more than 50% of the main stems had extended their final leaf by 1–2 cm, half of the plants were exposed to high-temperature (HT) conditions as the treatment group, while the other half were maintained under normal temperature as the control group. After 12 days of artificial high-temperature treatment, the heat-treated plants were returned to normal temperature.

At maturity, the seed-setting rates of all labeled panicles were recorded. The control group provides a baseline estimate of seed-setting capacity under normal conditions in an artificial climate chamber (Figure 5, lower panel). If no significant differences were observed among the tested varieties in the control group, heat tolerance can be directly compared using the seed-setting rates of the treatment group. Otherwise, a heat-tolerance index of each tested line can be calculated using the formula as “1 − (*SR_RT_* − *SR_HT_*)/*SR_RT_*”, where *SR_RT_* and *SR_HT_* represent the seed-setting rates under room temperature (RT) and high temperature (HT), respectively. This enables accurate evaluation of varietal differences in high-temperature tolerance during gametogenesis.

### 2.4. Validation of the Evaluation System Using Heat-Tolerant and Heat-Sensitive Varieties

**Figure 6:**
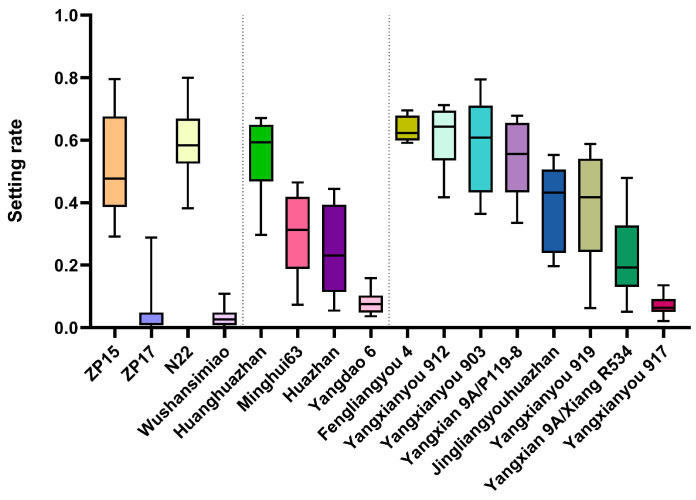
Identification of heat resistance of different cultivars. The horizontal axis represents different varieties, and the vertical axis denotes the seed-setting rate. Values are presented as mean ± SD. These varieties are divided into three groups by a dotted line: the left group contains two controls and two test lines, the middle group consists of four conventional rice varieties, and the right group is composed of eight hybrid rice varieties. Each group contains 6 pots for each tested line.

To validate the reliability of this system, we tested two representative rice varieties: a widely recognized heat-tolerant variety Nagina 22 (N22) and a heat-sensitive variety Wushansimiao (WSSM) commonly used in production. In a normal growth chamber, both exhibited normal seed-setting rates, with no significant difference detected between them. In contrast, after heat treatment, N22 maintained a seed-setting rate of 59.54%, whereas WSSM dropped sharply to 3.16% (Table 1, Figure 6 left panel). These results confirmed that the system accurately distinguishes the heat-tolerant and heat-sensitive varieties at the pollen developmental stage.

### 2.5. Evaluation of Heat Tolerance in Four Rice Varieties and Eight Hybrids

Using this evaluation system, we further assessed the high-temperature tolerance of four conventional varieties and eight hybrids. The four tested varieties, namely Huanghuazhan, Minghui 63, Huazhan and Yangdao 6, showed no significant difference from the control variety N22 under normal growth conditions. Under heat stress, however, their seed-setting rates varied markedly (Table 1, middle panel of Figure 6). Huanghuazhan exhibited the highest heat tolerance, maintaining a seed-setting rate of 55.96%, followed by Minghui 63 (29.13%) and Huazhan (24.10%). In contrast, Yangdao 6 (9311) showed the lowest seed-setting rate (7.99%), indicating particularly poor heat tolerance and a clear requirement for genetic improvement.

Among the eight hybrids, Fengliangyou 4 exhibited the highest seed-setting rate under heat stress (63.48%). Additionally, three self-bred hybrids—Yangxianyou 912, Yangxianyou 903, and Yangxian 9A/P119-8—also showed high heat tolerance comparable to that of Fengliangyou 4. In contrast, the remaining hybrids displayed insufficient heat tolerance, as their seed-setting rates were significantly lower than those of Fengliangyou 4; notably, Yangxianyou 917 had the lowest rate at only 7.08% (Table 1, right panel of Figure 6). These results demonstrate that our evaluation system can reliably differentiate high-temperature tolerance levels across both conventional varieties and hybrids during the pollen developmental stage.

## 3. Discussion

In general rice production, it is difficult to compare heat tolerance among varieties due to variation in growth duration and development, especially for the pollen developmental stage. This underscores the urgent need for a precise and feasible evaluation system. ZP15 and ZP17 are intermediate breeding materials derived from the same genetic background, Yangdao6 (9311). Whether grown in paddy fields or artificial climate chambers, they exhibit similar heading dates, panicle numbers, and plant height. Resequencing revealed SNP variation in only 13 small chromosomal regions (Supplemental Figure S4), suggesting that these lines behave as near-isogenic lines. Their synchronized heading facilitated the direct observation of contrasting heat tolerance during a natural heat event in 2017, which was subsequently verified under controlled high-temperature treatment. ZP15 and ZP17 provided excellent controls for the establishment of this system.

### 3.1. Divergence in Heat Tolerance Between ZP15 and ZP17 Is Concentrated in the Gametogenesis/Pollen Developmental Stage

Heat treatment at the seedling stage revealed no clear differences, but significant divergence emerged at the reproductive stage. Pollen development and viability was notably more tolerant in ZP15 than in ZP17 under high temperature, as demonstrated by iodine and TTC staining (Figure 3). We also cytologically observed pollen development in the two lines under high temperature and found that the tapetal degradation process was significantly affected in ZP17 under heat stress, but not in ZP15. These results are consistent with the observed differences in final seed-setting rates, suggesting that the higher heat tolerance of ZP15 is attributed to greater tolerance during its pollen developmental stage. Staggered sowing confirmed that only panicles exposed to high temperature from 9 to 16 days before heading showed remarkable differences between ZP15 and ZP17. According to the growth characteristics of Yangdao6 [20], this period corresponds precisely to gametogenesis, the most heat-sensitive phase in rice reproduction. Compared with ZP17, the higher heat tolerance of ZP15 is primarily attributable to the resilience in pollen development during gametogenesis.

### 3.2. A Simplified Evaluation System with High Accuracy and Broad Applicability

Guided by ZP15 and ZP17, we established an evaluation system for heat tolerance targeting gametogenesis. The inclusion of a prediction group allowed accurate identification of the developmental stage. To accurately identify the onset of pollen development, we referred to the research of Prof. Wang [20]. At the initiation of pollen development, the leaf age remainder is 0.8–0.9, and the spikelet length is 1.0–1.9 mm. We combined both criteria to make accurate predictions. Also, the appropriate timing of heat treatment and sampling was determined according to the data of ZP15 and ZP17 presented in the Supplementary Figure S1. Previous studies have reported a critical temperature threshold of ~34 °C at late developmental stages [21]. We adopted a constant 35 °C during the day and 29 °C at night, conditions more rigorous than typical field scenarios, where daily peaks often exceed 35 °C but not continuously. Preliminary trials conducted at constant temperatures of 38 °C and 41 °C resulted in severe or complete heading failure, along with poor fertility, in both ZP15 and ZP17 (Supplemental Figure S2), confirming that the 35/29 °C regime represents stringent stress. As panicle differentiation and the subsequent reproductive growth patterns are relatively conserved in rice, the duration from panicle differentiation to heading is approximately 30 days in most varieties, with the gametophyte and pollen development stage lasting around 12 days. We therefore set the heat stress duration to 12 days to cover the entire pollen developmental process. The conserved developmental rhythm provides a unified physiological basis for the application of our system to a wide range of rice accessions, including indica and japonica cultivars as well as locally adapted germplasm with distinct genetic backgrounds. Although minor variations in pollen developmental timing may exist among different genotypes, the system can be flexibly adapted by appropriately adjusting the initiation of high-temperature treatment according to the actual developmental stage of the target material, ensuring that heat stress is precisely imposed during the critical pollen development period. Therefore, the system is not restricted to the two near-isogenic lines ZP15 and ZP17 used for its establishment, but can serve as a reliable and convenient reference framework for evaluating heat tolerance during pollen development in diverse rice materials. Future studies may further validate and optimize this system using larger and more genetically diverse rice panels to enhance its universality and practical value in heat tolerance breeding programs.

Unlike conventional approaches that often treat the whole plant or broad developmental windows [16,19], our system focuses on discrete developmental stages, thus improving the accuracy and resolution of heat stress assessment. The reliability of this evaluation system was first verified with N22 and WSSM, and subsequently confirmed by the graded tolerance responses exhibited by four conventional varieties and eight hybrids. Collectively, these findings confirm that the system exhibits high accuracy in differentiating between heat-tolerant and heat-sensitive genotypes. The method established in the present study allows for a more precise and stage-specific manipulation and evaluation of heat responses during pollen development.

### 3.3. Implications for Breeding and Germplasm Improvement

ZP15, identified as an intermediate breeding line, exhibited heat tolerance comparable to that of N22, making it a valuable source of heat-resistant germplasm. Given its genetic background—Yangdao 6 (9311) is highly sensitive to heat during gametogenesis—improving the heat tolerance of Yangdao 6 is critical for its sustained utilization in production. ZP15 holds potential both as an alternative to Yangdao 6 and as a donor parent for genetic improvement of heat tolerance. Consistent with previous reports, Huanghuazhan also displayed strong heat tolerance, highlighting its potential as another important resource for heat-tolerant rice breeding [17]. The identification of heat-sensitive restorer lines such as Minghui 63 and Huazhan has important practical implications for hybrid rice breeding and production. As these elite restorer lines are widely used in hybrid rice combinations, clarifying their heat sensitivity during pollen development provides a critical reference for hybrid seed production. This knowledge allows breeders to optimize sowing dates, adjust field management practices, and implement targeted cultivation strategies to avoid high-temperature damage during the most vulnerable reproductive stages. Furthermore, these heat-sensitive materials can serve as valuable controls for evaluating heat tolerance in breeding programs, facilitating the selection and breeding of new heat-resistant restorer lines to ensure a stable yield and quality of hybrid rice under climate change. These results also highlight the necessity of enhancing heat tolerance in major restorer lines to ensure the stability of hybrid rice production under global warming scenarios.

Although hybrids are generally associated with heterosis (hybrid vigor), significant variation in heat tolerance was observed among the eight tested hybrids. Fengliangyou 4 and Yangxianyou 912 exhibited the highest heat tolerance, while Yangxianyou 917 was highly heat-sensitive. Notably, the heat tolerance of the hybrids was closely correlated with that of their respective restorer lines: Yangxianyou 912 showed heat tolerance like ZP15, Jingliangyouhuazhan resembled Huazhan, and Yangxianyou 917 matched Yangdao 6. This suggests that restorer lines play a dominant role in regulating heat tolerance during the gamete stage in hybrid rice. Thus, prioritizing the improvement of restorer line heat tolerance should be a key strategy to enhance hybrid performance under heat stress. Furthermore, although less extensively studied, strengthening pistil tolerance in male sterile lines may stabilize female gametes, facilitate fertilization, and improve embryo development under heat stress. Our evaluation system covers not only pollen development but also pistil development; therefore, it can also be utilized in future studies to assess the high-temperature tolerance of pistils in male sterile lines.

### 3.4. A Platform for Genetic Studies of Heat Tolerance

Although several genes, including *OgTT1*, *TT2*, *TT3.1*, *TT3.2*, *SLG1*, and *HTH5* [22,23,24,25,26], have been identified as key regulators of heat tolerance in rice, it remains critical to identify additional genes to fully elucidate the genetic basis of heat tolerance, particularly during pollen development. In this study, ZP15 and ZP17 are a pair of near-isogenic lines with highly similar flowering times. Furthermore, they exhibited stable and extremely significant differences in heat tolerance based on our evaluation system, which provides a solid foundation for dissecting the genetic differences between them. Indeed, we have already identified a novel QTL using the population derived from these two lines, and related research is currently underway. Integrating our system with forward genetics, transcriptomic profiling, and gene functional validation will provide powerful opportunities to pinpoint key genes and molecular mechanisms underlying thermotolerance in pollen development stages. Such efforts are not only essential for unraveling the regulatory networks governing stress responses but also hold great promise for improving crop resilience under challenging environmental conditions. Therefore, combining this system with in-depth molecular characterization will form a major focus of our future investigations. This work provides a solid foundation and an excellent platform for dissecting the genetic basis of heat tolerance.

## 4. Materials and Methods

### 4.1. Plant Materials

Two lines, ZP15 and ZP17, were selected in breeding by Professor Li Aihong and Xiao Ning at Lixiahe agricultural institute of Yangzhou. Both were derived from indica rice variety Yangdao 6 (alternatively named 9311) (Supplemental Figure S5). They showed divergent heat tolerance under natural high-temperature conditions in 2017. Since the difference between them was found focusing on the gamete development stage, we used them as references to establish the evaluation system.

The system was further verified by two rice varieties with well-characterized heat tolerance, including heat-tolerant variety N22 and heat-sensitive variety Wushansimiao, in rice production [18,19]. Moreover, four conventional rice varieties, Huanghuazhan, Huazhan, Yangdao6, Minghui 63, and two control hybrids, Fengliangyou4 and Jingliangyouhuazhan, as well as six our self-bred hybrids, Yangxianyou912, Yangxianyou903, Yangxian 9A/P119-8, Yangxianyou919, Yangxian 9A/Xiang R534 and Yangxianyou917, were tested in this system.

### 4.2. Field Planting

For the propagation and agronomic trait investigations, all the materials were grown in the paddy field of Yangzhou University. The seeds were sown around 12 May and the seedlings were transplanted on 12 June. Each material was planted in rows with a spacing of 13.3 cm and the interval between rows was 26.3 cm. Water and fertilizer management was carried out according to the conventional cultivation method for rice.

### 4.3. Agronomic Trait Investigation

Several agronomic traits were investigated to compare ZP15 and ZP17 under both normal and high-temperature conditions. When a panicle extended 1–2 cm out of the sword leaf, the day was recorded as the heading day of the panicle. The day number between the heading and sowing period is calculated as the heading date. To calculate the heading date of each line, we only investigated the main panicle of one plant and at least 10 individuals were investigated. Spikelet fertility was estimated by the seed-setting rate, defined as the percentage of fully filled seeds per panicle. The panicles that met the effective treatment requirements were labeled randomly. For each line, the seed-setting rate value represents the mean across all labeled panicles from all replicate plants in pots. When the plants were maturing, the plant height was measured from the bottom to the top. The tillers with fertile spikelets were counted as effective tillers. All the data were represented as mean ± standard deviation.

### 4.4. Potted Cultivation

For potted cultivation, seeds were soaked in running water for about 24 h and followed by accelerating germination at 30 °C for 12 h in an incubator. When the bud appeared, the seeds were sown in plastic trays. Subsequently, 28-day-old seedlings were transplanted into large plastic pots (25 cm in diameter and 30 cm in height) filled with natural clay loam soil. Each pot contains three to four plantlets, and at least 6 pots are needed in each group for each tested line. The pots were randomly arranged in a net-house and the plants were grown under natural temperature and sunlight until the treatment begins.

### 4.5. Artificial Temperature Treatments

For the normal temperature treatment, the growth chambers were set at a constant 28 °C in the day time and 22 °C at night. For the high-temperature treatment, the chamber was set at a constant 35 °C in the day time and 29 °C at night. We used BIOVOICE Temperature Dataloggers, T301 (Dianjing New Materials company, Nantong, China), to monitor the actual temperatures in each growth chamber every 3 min (as shown in Supplemental Figure S3). The light source used in the growth chamber was a white LED light (60,000 LUX light intensity) with a photoperiod of 14 h light/8 h dark. The relative humidity was controlled at 70% throughout the experiment. To identify the critical window of heat responding divergence between ZP15 and ZP17, five successive sowings on April 19, 22, 25, and 28 and May 1 were conducted in Yangzhou summer season. About 100 seeds were used for each line in each batch. On May 21, 180 seedlings from each line were transplanted to potted cultivation. On 7 July, when a small portion of panicles commenced heading, all pots were moved into an indoor growth chamber (ESHENGTAIHE, Beijing, China) and subjected to a 12-day heat treatment.

### 4.6. Iodine–Potassium Iodide (I_2_–KI) Staining Assay

Both ZP15 and ZP17 panicles that flowered within 7 days after the high-temperature treatment were collected. For each panicle, the viability of pollen in at least 10 florets was evaluated using the I_2_–KI staining assay. The I_2_–KI staining solution (1% I_2_ and 8% KI) was prepared and reserved in a brown bottle at 4 °C for long-term storage. Before staining, a drop of I_2_–KI solution was loaded on the glass slide. Fully developed anthers were collected and clamped by surgical forceps in the solution. After removing the debris, the slide was covered carefully by a coverslip and imaged under a light microscope (Leica DM2500 Led,,Wetzlar, Germany). For each floret, pollen grains were observed under a 10× magnification lens, and more than 5 photos were captured from different fields of view, with 300–500 pollen grains per image. To simplify the results, we manually evaluated the proportion of deeply stained pollen grains for each floret and classified all florets into four categories based on their staining rates: ≤10%, 10–50%, 50–80%, and ≥80%.

### 4.7. TTC Assay of Pollen

The viability of pollen was evaluated by 2,3,5-triphenyl-tetrazolium chloride (TTC) reduction assay. First, two drops of 1% TTC solution (CAS T395987, Aladdin, Shanghai, China) were loaded on the glass slide. Anthers from the florets were put into the solution and clamped by surgical forceps to make sure that as much pollen as possible was released and totally soaked in the TTC solution. The slides were then incubated at 37 °C for 0.5 h in the dark. The stained pollen was observed under a light microscope (Leica DM2500 Led, Wetzlar, Germany) and imaged with 100–200 pollen grains under a 10× magnification lens. The viability was represented by the tinting degree or the percentage of deeply stained pollens.

### 4.8. Statistical Analysis

All data are expressed as the mean ± standard deviation (SD). All significance between data was statistically analyzed and compared using a two-tailed heteroscedastic *t*-test (Welch’s *t*-test) in Excel software. This test was employed without assuming equal variances between the compared groups, and significance was determined based on the resulting *p*-values. ZP17 was compared with ZP15 in our preliminary experiments and formal experiments. N22 was taken as a control for all the conventional varieties and Fengliangyou 4 was taken as a control for all the hybrids.

## 5. Conclusions

Based on the distinct heat tolerance divergence between ZP15 and ZP17, we established an accurate and feasible evaluation system. This system provides a valuable tool to assess heat tolerance during the critical pollen development period. By fine-tuning the heat treatment window, it can be applied in pre- or post-pollen developmental stages. This system can be effectively integrated into DNA mapping and molecular breeding to maximize its practical value. For DNA mapping, the system can be used to precisely phenotype large segregating populations (e.g., F2, RILs) derived from heat-tolerant and heat-sensitive parents (such as ZP15 and ZP17), generating reliable phenotypic data that can be combined with genotypic data for quantitative trait locus (QTL) mapping or genome-wide association studies (GWASs). For molecular breeding, the system can serve as a core phenotypic evaluation tool to screen heat-tolerant germplasm at early generation, accelerate the selection process of heat-resilient rice varieties, and reduce the time and cost of traditional breeding. Therefore, it will accelerate the genetic improvement of rice heat tolerance, facilitating the development of resilient cultivars to address the challenges of climate change.

Despite its considerable application potential, its applicability to a wider range of rice germplasms, especially those with noticeably shifted pollen developmental timing, remains to be further verified. The optimization of heat treatment parameters (e.g., temperature duration, intensity gradient) for different rice ecotypes needs to be further refined to enhance the system’s universality.

## Figures and Tables

**Table 1:** Information of rice varieties tested by the evaluation system.

Type	Name	HT Time	Label Time	RT-SR	HT-SR	SD	*p*-Value
Controls	ZP15 *	7/07–7/18	7/16–7/22	84.56%	50.51%	0.15	/
	ZP17	7/07–7/18	7/16–7/22	85.84%	4.25%	0.07	<0.0001
Test lines	N22 *	8/11–8/22	8/20–8/25	86.20%	59.54%	0.11	/
	Wushansimiao	8/02–8/13	8/11–8/16	83.17%	3.16%	0.03	<0.0001
Conventional rice varieties	Huanghuazhan	7/27–8/08	8/06–8/11	82.31%	55.96%	0.12	0.44
	Minghui 63	8/04–8/17	8/14–8/20	84.67%	29.13%	0.13	<0.0001
	Huazhan	7/30–8/11	8/08–8/14	81.26%	24.10%	0.14	<0.0001
	Yangdao 6	8/05–8/18	8/15–8/21	80.33%	7.99%	0.04	<0.0001
Hybrid rice varieties	Fengliangyou 4 *	8/07–8/18	8/16–8/21	85.71%	63.48%	0.04	/
	Yangxianyou 912	7/31–8/11	8/09–8/14	83.57%	61.33%	0.11	0.61
	Yangxianyou 903	7/31–8/11	8/09–8/14	84.15%	58.66%	0.15	0.39
	Yangxian 9A/P119-8	7/31–8/11	8/09–8/14	86.09%	54.08%	0.12	0.05
	Jingliangyouhuazhan	8/06–8/17	8/15–8/20	82.65%	39.32%	0.13	<0.0001
	Yangxianyou 919	8/09–8/20	8/18–8/23	84.49%	37.05%	0.19	0.0013
	Yangxian 9A/Xiang R534	7/31–8/11	8/09–8/14	81.78%	22.38%	0.13	<0.0001
	Yangxianyou 917	8/04–8/15	8/13–8/18	82.33%	7.08%	0.03	<0.0001

*: Statistical references. RT-SR indicates setting rates at room temperature; HT-SR indicates setting rates at high-temperature treatment; SD indicates standard deviation at HT. ZP17 was compared with ZP15; Wushansimiao and the other four conventional varieties were compared with N22; all hybrids were compared with Fengliangyou 4.

## Data Availability

The original contributions presented in this study are included in the article/Supplementary Material. Further inquiries can be directed to the corresponding authors.

## Supplementary Materials

The following supporting information can be downloaded at: https://www.mdpi.com/article/10.3390/plants15081253/s1, Figure S1: The pedigree information of ZP15 and ZP17; Figure S2: Real-time monitoring of temperature in high-temperature treatment chamber; Figure S3: Identification of the critical window for heat-tolerance divergence between ZP15 and ZP17; Figure S4: SNP distribution between ZP17 and ZP15. Figure S5: The pedigree information of ZP15 and ZP17.

## Abbreviations

The following abbreviations are used in this manuscript:

I_2_–KI	iodine–potassium iodide
TTC	triphenyltetrazolium chloride
N22	Nagina 22
WSSM	Wushansimiao
HD	heading date
HT	high temperature
RT	room temperature
