# Supplementary material for: A Simplified Heat-Tolerance Evaluation System at the Pollen Development Stage in Rice (Oryza sativa L.)
Source: Plants (Basel). 2026 Apr 18;15(8):1253. doi: 10.3390/plants15081253 (PMC13120345; doi:10.3390/plants15081253)
Supplement: Supplementary file 1 [file plants-15-01253-s001.zip › plants-4213018-supplementary.pdf]

No. of treament days	HD	P-value	Days before heading																							heading days							
			-23	-22	-21	-20	-19	-18	-17	-16	-15	-14	-13	-12	-11	-10	-9	-8	-7	-6	-5	-4	-3	-2	-1	1	2	3	4	5	6	7	8
6th	7.12	0.629468671																															
7th	7.13	0.048529052																															
8th	7.14	0.377040072																															
9th	7.15	0.170365733																															
10th	**7.16**	1.67858E-06																															
11th	**7.17**	3.85596E-19																															
12th	**7.18**	5.30115E-12																															
13th	**7.19**	2.85967E-05																															
14th	**7.20**	1.52867E-05																															
15th	**7.21**	9.16648E-05																															
16th	**7.22**	0.000182265																															
17th	**7.23**	0.000170009																															
18th	7.24	0.066127906																															
19th	7.25	0.046500101																															
20th	7.26	0.333402343																															
21st	7.27	0.799041826																															
22nd	7.28	0.093964777																															
23rd	7.29	0.127654453																															

Orange region: 12-days Heat treatment (7.7-7.18)

Green region: Critical window

Supplemental Figure S1 Identification of the critical window for heat-tolerance divergence between ZP15 and ZP17

A 12-day treatment was conducted from July 7 to 18 in 2017 and the period was indicated in orange. The heading panicles were labelled from July 12 to 29. The seed-setting rates of the panicles heading from July 16 to 23 showed significances between ZP15 and ZP17 (marked in yellow), which suggests that the critical window (marked in green) for their divergence is 9 to 16 days before heading.

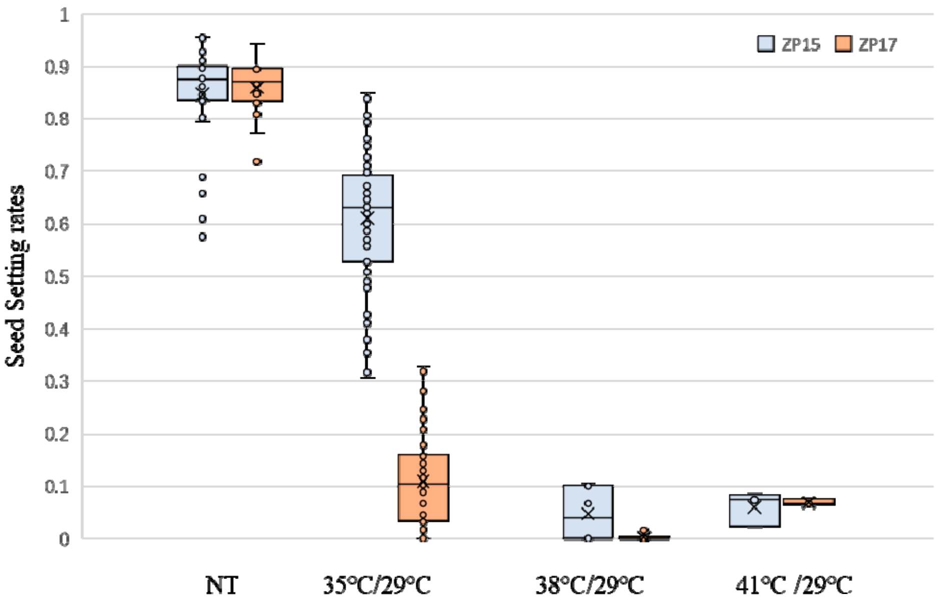

Supplemental Figure S2 Temperature tests of the evaluation system using ZP15 and ZP17. The x-axis indicates different temperature treatments: NT, normal temperature; 35℃/29℃, 38℃/29℃ and 41℃/29℃ represent different temperature treatments, with daytime temperatures maintained at a constant 35℃, 38℃ and 41℃, respectively, and nighttime temperature maintained at a constant 29℃. The y-axis represents the seed-setting rates. At normal temperature, both ZP15 and ZP17 exhibited normal fertility. At 35℃/29℃, panicles sampled on specific days (data from July 17–19) showed significant differences between the two materials. At 38℃/29℃ and 41℃/29℃, the heading process was hindered and delayed. The final heading panicles (data from July 27 to August 1) still exhibited near-sterility.

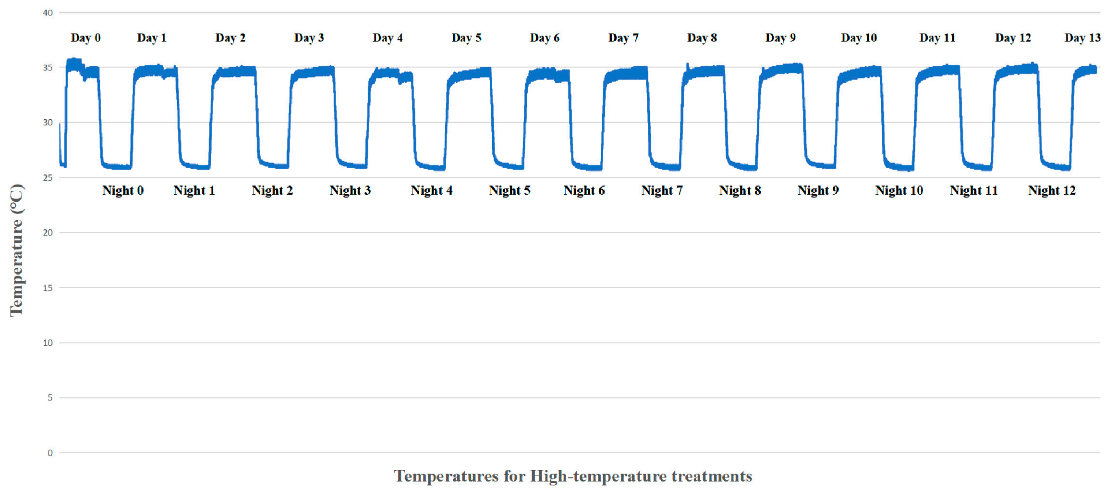

Supplemental Figure S3 Real-time monitoring of temperature in high-temperature treatment chamber

Day 0 is the preparation day before treatment. High-temperature treatment was applied from Day 1 to Day 12. Day 13 is the final day when samples were removed.

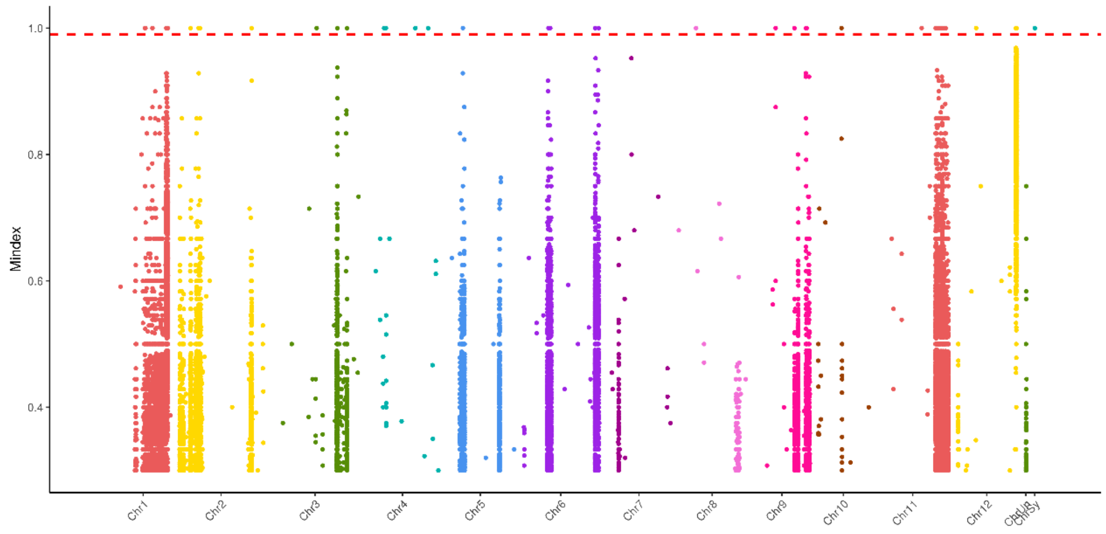

Supplemental Figure S4 SNP distribution between ZP17 and ZP15

Colored dots represent SNP loci. The x-axis indicates different chromosomal positions. From their derived F2 population, the high-temperature sensitive individuals were sampled as a pool and sequenced. The y-axis (Mindex) represents the frequency of a given SNP in the pool.

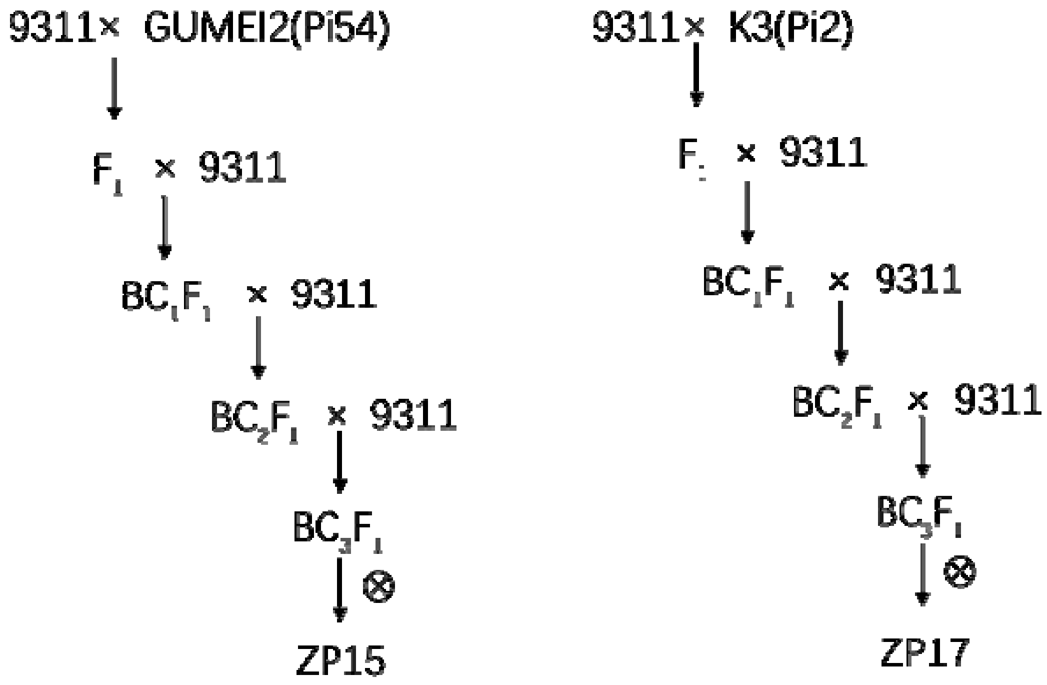

Supplemental Figure S5 The pedigree information of ZP15 and ZP17

ZP15 was selected from the BC_3 progenies derived from the cross between 9311 and GUMEI2 (carrying the Pi54 locus). This line possesses blast resistance conferred by Pi54 under 9311 genetic background and shows strong high-temperature tolerance.

ZP17 was selected from the BC_3 progenies derived from the cross between 9311 and K3 (carrying the Pi2 locus). This line possesses blast resistance conferred by Pi2 under 9311 genetic backgrounds but shows weak high-temperature tolerance.
